# Tranexamic acid blocks the thrombin-mediated delay of epidermal permeability barrier recovery induced by the cedar pollen allergen, Cry j1

**DOI:** 10.1038/s41598-018-33898-7

**Published:** 2018-10-23

**Authors:** S. Nakanishi, J. Kumamoto, M. Denda

**Affiliations:** 1 Shiseido Research Center, Yokohama, Japan; 2 Research Institute for Electronic Science, Hokkaido University, Sapporo, Japan

## Abstract

We previously demonstrated that Cry j1, the major pollen allergen of *Cryptomeria japonica* (Japanese cedar), transiently increases protease activity and intracellular Ca^2+^ concentration in cultured human keratinocytes, and delays recovery after stratum corneum barrier disruption in human skin *ex vivo*. Topical application of tranexamic acid or trypsin-type serine protease inhibitors accelerates barrier recovery. We hypothesized that tranexamic acid might prevent the transient protease activity increase and the barrier recovery delay induced by Cry j1. Here, we tested this hypothesis and examined the mechanism involved. In cultured human keratinocytes, knock-down of protease-activated receptor 1 (PAR-1) reduced the transient increase of calcium induced by Cry j1, whereas knock-down of PAR-2 did not. Knock-down of thrombin significantly reduced the transient increases of calcium concentration and protease activity. Tranexamic acid, soybean trypsin inhibitor, or bivalirudin (a thrombin inhibitor) also reduced the calcium elevation induced by Cry j1 and/or thrombin. Co-application of tranexamic acid or bivalirudin with Cry j1 to human skin *ex vivo* blocked the delay of barrier recovery. These results suggest that thrombin and PAR-1 or PAR-1-like receptor might mediate the adverse effects of Cry j1 on human epidermal keratinocytes, and could open up a new strategy for treating inflammatory skin diseases.

## Introduction

We previously demonstrated that the major Japanese cedar (*Cryptomeria japonica*) pollen allergen, Cry j1, perturbs epidermal permeability barrier recovery after disruption^1^. We also observed a transient increase of protease activity and an elevation of intracellular calcium level in keratinocytes incubated with Cry j1. In addition, inhibitors of trypsin-type serine protease and protease-activated receptor 2 (PAR-2) reduced the calcium elevation, and topical application of the inhibitors after barrier disruption in an *ex-vivo* human skin system decreased the Cry j1-induced delay of the barrier recovery^1^. We have also reported that topical application of tranexamic acid after barrier disruption accelerated the barrier recovery^2^. Furthermore, protease activity was transiently increased in the epidermis after barrier disruption, and tranexamic acid partially blocked this increase. Based on these observations, we hypothesized that application of tranexamic acid might prevent the increase of protease activity and reduce the delay of barrier recovery induced by Cry j1.

The aim of the present study was to test this hypothesis and to examine the mechanism involved. It is well known that tranexamic acid inhibits protease activity by blocking the activation of plasminogen to plasmin, a serine protease^3,4^. However, we found here that plasmin/plasminogen expression is undetectable in keratinocytes, and instead, tranexamic acid appears to block the adverse effects of Cry j1 on keratinocytes and epidermal permeability barrier homeostasis via a pathway involving thrombin and protease-activated receptor 1 (PAR-1) or a PAR-1-like receptor. Thus, thrombin and PAR-1 or the PAR-1-like receptor may be available as new therapeutic targets to improve epidermal barrier homeostasis in the presence of harmful environmental factors such as Cry j1.

## Results

### Transcript-level analysis of plasminogen and plasmin in human keratinocytes

Based on previous studies showing that urokinase-type plasminogen activator is associated with barrier function in human skin^5,6^ and that tranexamic acid, a plasminogen inhibitor, accelerates skin barrier recovery^2,7^, we initially expected that the impairment of human skin barrier function by Cry j1 would be due to plasminogen activation. Therefore, we examined the expression of plasminogen and plasmin by RT-PCR. However, we detected no expression of plasminogen or plasmin in cultured human keratinocytes (data not shown). This result suggests that some other serine protease(s) is involved in the Cry j1-induced impairment of barrier function.

### Functional relationship of PAR-1 and PAR-2 to Cry j1-induced calcium elevation

**Figure 1:**
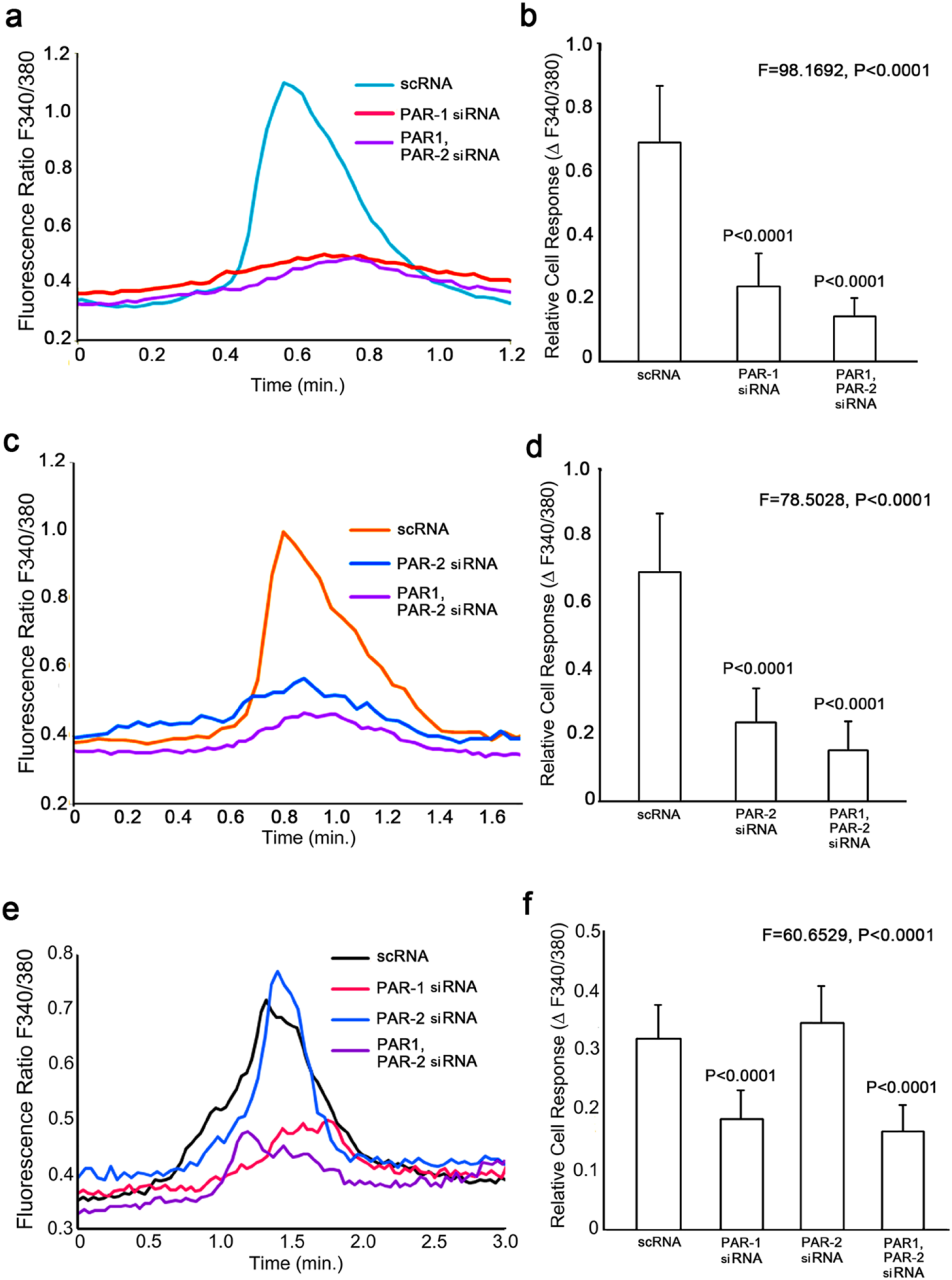
Calcium response induced by PAR-1 agonist, PAR-2 agonist or Cry j1 in PAR-1 and PAR-2 siRNA-treated keratinocytes. (**a**) Application of TFLLR-NH2 (10 μM) to the scramble RNA-treated cells increased the level of intracellular calcium by approximately 0.69 unit (cyan line), while application of the same amount of TFLLR-NH2 to the PAR-1 siRNA-treated cells (red line) and PAR-1 plus PAR-2 siRNA-treated cells (purple line) increased the level of intracellular calcium by approximately 0.24 unit and 0.14 unit, respectively. (**b**) Quantitation of fluorescence ratio change after application of TFLLR-NH2 to cells treated with scramble RNA, PAR-1 siRNA and PAR-2 siRNA (n = 11–20 cells). Similar results were obtained in three independent experiments. Bars and lines represent mean ± SD. (**c**) Application of SLIGKV-NH2 (10 μM) to the scramble RNA-treated cells increased the level of intracellular calcium by approximately 0.69 unit (cyan line), while application of the same amount of SLIGKV-NH2 to the PAR-1 siRNA-treated cells (red line) and PAR-1 plus PAR-2 siRNA-treated cells (purple line) increased the level of intracellular calcium by approximately 0.24 unit and 0.15 unit, respectively. (**d**) Quantitation of fluorescence ratio change after application of SLIGKV-NH2 to cells treated with scramble RNA, PAR-1 siRNA and PAR-2 siRNA (n = 11–20 cells). Similar results were obtained in three independent experiments. Bars and lines represent mean ± SD. (**e**) Application of Cry j1 (100 ng/ml) to the scramble RNA-treated cells increased the level of intracellular calcium by approximately 0.32 unit (black line), while application of the same amount of Cry j1 to the PAR-1 siRNA-treated cells (red line) and PAR-1 plus PAR-2 siRNA-treated cells (purple line) increased the level of intracellular calcium by approximately 0.18 unit and 0.16 unit, respectively. Application of the same amount of Cry j1 to the PAR-2 siRNA-treated cells increased the level of intracellular calcium by approximately 0.34 unit (blue line). (**f**), Quantitation of fluorescence ratio change after application of Cry j1 to cells treated with scramble RNA, PAR-1 siRNA and PAR-2 siRNA (n = 20 cells). Similar results were obtained in three independent experiments. Bars and lines represent mean ± SD.

To examine the involvement of protease-activated receptors in the Cry j1-induced calcium elevation, we performed knock-down studies with siRNAs targeting PAR-1, -2, -3 and -4. siRNA functionality was verified by RT-PCR and by measuring the calcium response to specific agonists (PAR-1 agonist: TFLLR-NH2, PAR-2 agonist: SLIGKV-NH2). RT-PCR revealed that PAR-1 expression was decreased by 89% and PAR-2 expression was decreased by 91% in the respective siRNA-treated keratinocytes (see Supplementary Fig. S1). PAR-1 down-regulation did not affect PAR-2 expression, and vice versa (see Supplementary Fig. S1). Keratinocytes co-treated with PAR-1 siRNA plus PAR-2 siRNA showed 89% and 76% reductions of PAR-1 and PAR-2 expression, respectively (see Supplementary Fig. S1). Application of TFLLR-NH2 to PAR-1 siRNA-treated and PAR-1 siRNA plus PAR-2 siRNA-treated keratinocytes resulted in a significantly lower calcium response: 34% and 20% of the control, respectively (Fig. 1a,b). Application of SLIGKV-NH2 to PAR-2 siRNA-treated and PAR-1 siRNA plus PAR-2 siRNA-treated keratinocytes also resulted in a significantly lower calcium response; 34% and 22% of the control, respectively (Fig. 1c,d). We could not verify PAR-3 and PAR-4 siRNA functionality, because the expression levels of PAR-3 and PAR-4 in untreated cultured human keratinocytes level were extremely low (data not shown).

In both PAR-1 siRNA-treated and PAR-1 siRNA plus PAR-2 siRNA-treated cells, the Cry j1-induced calcium elevations were significantly reduced (56% and 51% respectively), whereas treatment with PAR-2 siRNA alone had no effect (Fig. 1e,f). These results suggest PAR-1, but not PAR-2, might be a mediator of the Cry j1-induced calcium response.

### Functional relationship of thrombin to Cry j1-induced calcium elevation

**Figure 2:**
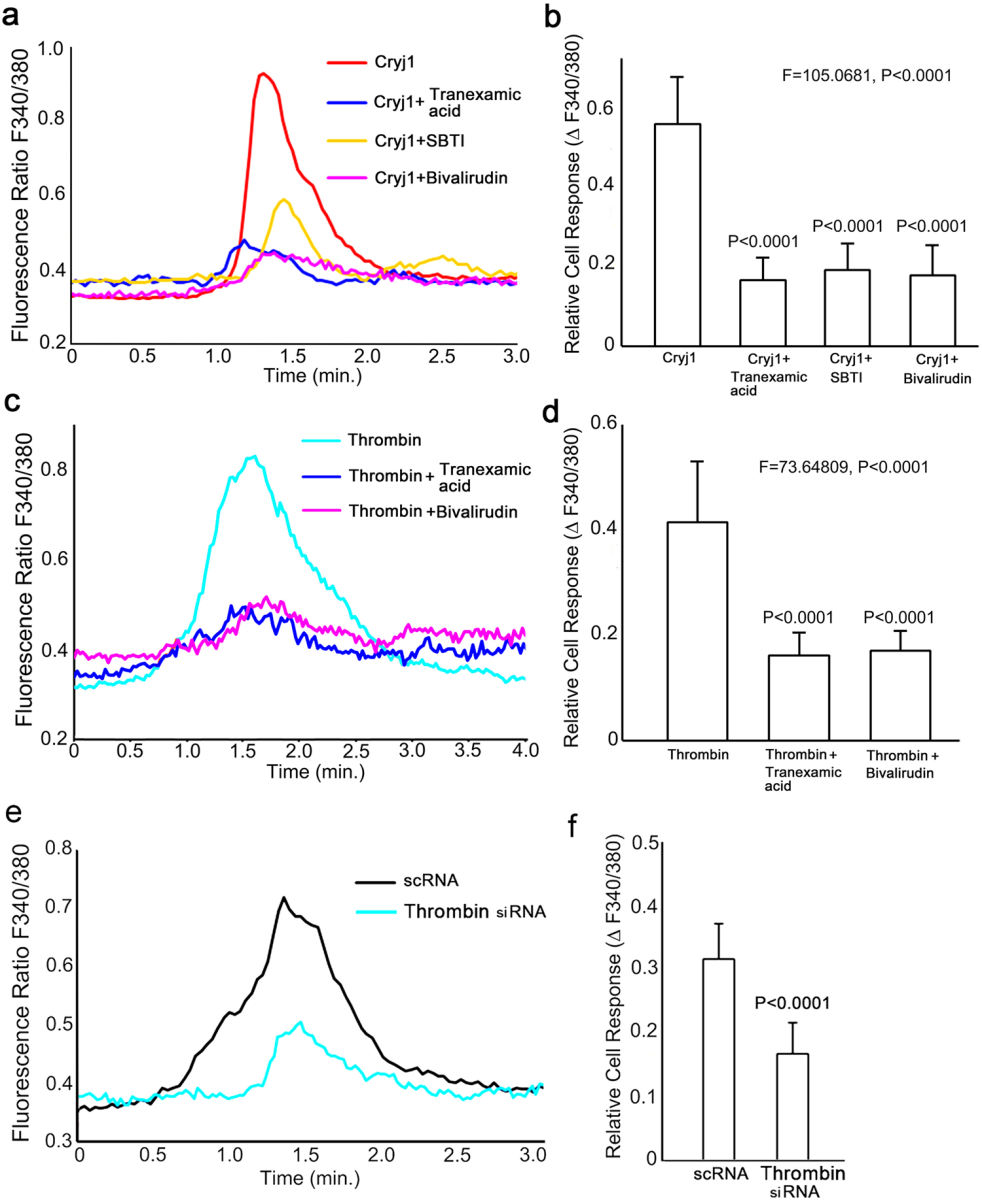
Calcium response of keratinocytes treated with Cry j1 or thrombin. (**a**) Application of Cry j1 (100 ng/ml) increased the level of intracellular calcium by approximately 0.56 unit (red line), while application of the same amount of Cry j1 together with tranexamic acid (10 mM) (blue line), SBTI (1 μM) (yellow line) or bivalirudin (100 ng/ml) (pink line) increased the level of intracellular calcium by approximately 0.16 unit, 0.19 unit and 0.18 unit, respectively. (**b**) Quantitation of fluorescence ratio change after application of Cry j1 with or without tranexamic acid, SBTI and bivalirudin (n = 20 cells). Similar results were obtained in three independent experiments. Bars and lines represent mean ± SD. (**c**) Application of thrombin (100 pg/ml) increased the level of intracellular calcium by approximately 0.41 unit (cyan line), while application of the same amount of thrombin with tranexamic acid (10 mM) (blue line) or bivalirudin (100 ng/ml) (purple line) increased the level of intracellular calcium by approximately 0.16 unit and 0.17 unit, respectively. (**d**) Quantitation of fluorescence ratio change after application of thrombin with or without tranexamic acid and bivalirudin (n = 20 cells). Similar results were obtained in three independent experiments. Bars and lines represent mean ± SD. (**e**) Application of Cry j1 (100 ng/ml) to scramble RNA-treated cells increased the level of intracellular calcium by approximately 0.32 unit (black line), while application of the same amount of Cry j1 to the thrombin siRNA-treated cells (cyan line) increased the level of intracellular calcium by approximately 0.17 unit. (**f**) Quantitation of the fluorescence ratio change after application of Cry j1 to the cells treated with scramble RNA and thrombin siRNA (n = 20 cells). Similar results were obtained in three independent experiments. Bars and lines represent mean ± SD.

Among the four types of PARs, PAR-2 is activated by trypsin-type serine protease, whereas the other PARs are activated by thrombin-type protease^8^. Based on the results of the siRNA study described above, we speculated that thrombin might be involved in Cry j1-induced calcium elevation, since it is expressed in human keratinocytes^9^. Thus, we examined the effect of bivalirudin, a thrombin inhibitor^10^ on the intracellular calcium response induced by Cry j1. Bivalirudin significantly reduced the Cry j1-induced intracellular calcium elevation (Fig. 2a,b). The effect of tranexamic acid was also assessed at 10 mM concentration, because it is reported that tranexamic acid prolongs the euglobulin clot lysis time at that concentration^11^. Although expression of plasminogen and plasmin in the cells was undetectable, we found that tranexamic acid nevertheless reduced the calcium elevation, as did soybean trypsin inhibitor (SBTI) (Fig. 2a,b).

Because thrombin is an activator of PAR-1, we next performed a calcium imaging experiment in the presence of thrombin. Application of thrombin to cultured keratinocytes elevated the intracellular calcium concentration and this elevation was significantly reduced to 39% by tranexamic acid and to 41% by bivalirudin (Fig. 2c,d). PAR-1 siRNA treatment, but not PAR-2 siRNA treatment, diminished the thrombin-induced calcium elevation, supporting the idea that the calcium elevation was mediated by PAR-1 (see Supplementary Fig. S2). We also examined the effect of knock-down of thrombin with siRNA on the intracellular calcium response induced by Cry j1. siRNA functionality was confirmed by ELISA, and the results revealed that the thrombin level was decreased by approximately 57% (P < 0.01) (control; 0.13 ± 0.048 ng/ml and thrombin siRNA-treated; 0.056 ± 0.035 ng/ml) in thrombin siRNA-treated keratinocytes. Thrombin siRNA-treated keratinocytes showed significantly lower Cry j1-induced intracellular calcium elevation (Fig. 2e,f).

**Figure 3:**
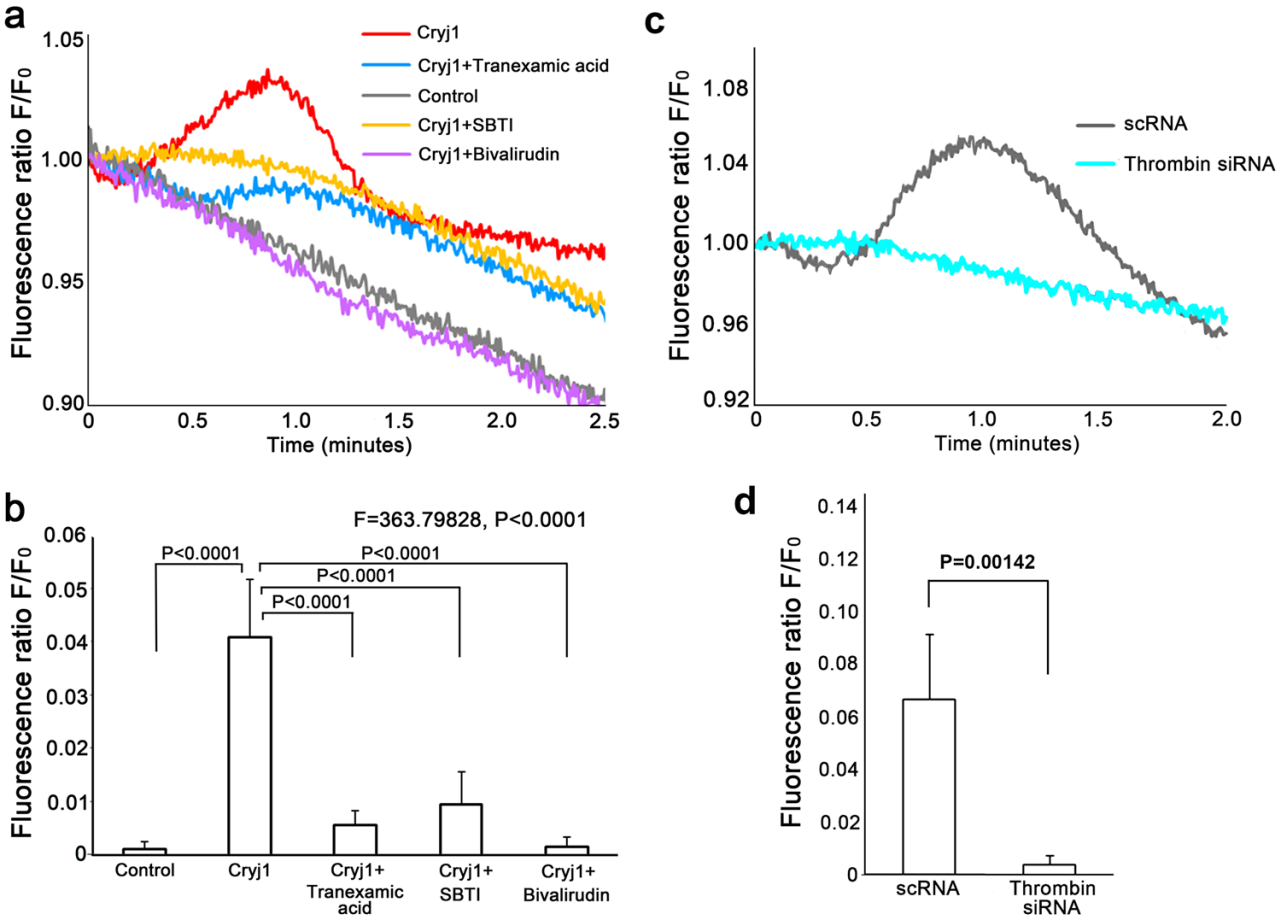
Protease activity of keratinocytes treated with Cry j1 with or without tranexamic acid, SBTI and bivalirudin. (**a**) Application of Cry j1 (100 ng/ml) to cultured human keratinocytes at time 0 s induced a rapid, transient increase of protease activity (red line). After application of BSS(+) alone (control), the fluorescence level gradually decreased (gray line). The transient increase was blocked by tranexamic acid (blue line), SBTI (yellow line) or bivalirudin (purple line). The vertical scale is normalized by the fluorescence at time 0. (**b**) Quantitation of fluorescence change within 1 min after application (n = 30–50 cells). A significant difference was observed between the control and Cry j1 application groups. Tranexamic acid, SBTI and bivalirudin each blocked the increase. Similar results were obtained in three independent experiments. Bars and lines represent mean ± SD. (**c**) Application of Cry j1 (100 ng/ml) to the cells treated with scramble RNA at time 0 s induced a rapid, transient increase of protease activity (gray line), while the application of the same amount of Cry j1 to the thrombin siRNA-treated cells induced a low transient increase (cyan line). The vertical scale is normalized by the fluorescence at time 0. (**d**) Quantitation of fluorescence change within 1 min after application (n = 30–50 cells). A significant difference was observed between the thrombin siRNA-treated cells and control. Similar results were obtained in three independent experiments. Bars and lines represent mean ± SD.

We next re-examined the induction of serine protease activity in keratinocytes by Cry j1 to confirm our previous findings^1^. Application of Cry j1 to cultured keratinocytes produced a transient increase of fluorescence (representing protease activity), which was independent of PAR-1 and PAR-2 (see Supplementary Fig. S3). The transient increase of fluorescence was blocked by bivalirudin, tranexamic acid and SBTI (Fig. 3a). The change of the fluorescence ratio within 1 min after application of Cry j1 is shown in Fig. 3b. The transient increase of protease activity induced by Cry j1 was significantly reduced to 12.5% by tranexamic acid, to 22.5% by SBTI, and to 3.75% by bivalirudin, compared to the control. It was also significantly reduced in thrombin siRNA-treated keratinocytes (Fig. 3c,d). These results support the idea that thrombin is involved in the Cry j1-induced calcium elevation.

### Comparison of epidermal permeability barrier recovery in an *ex-vivo* skin system

**Figure 4:**
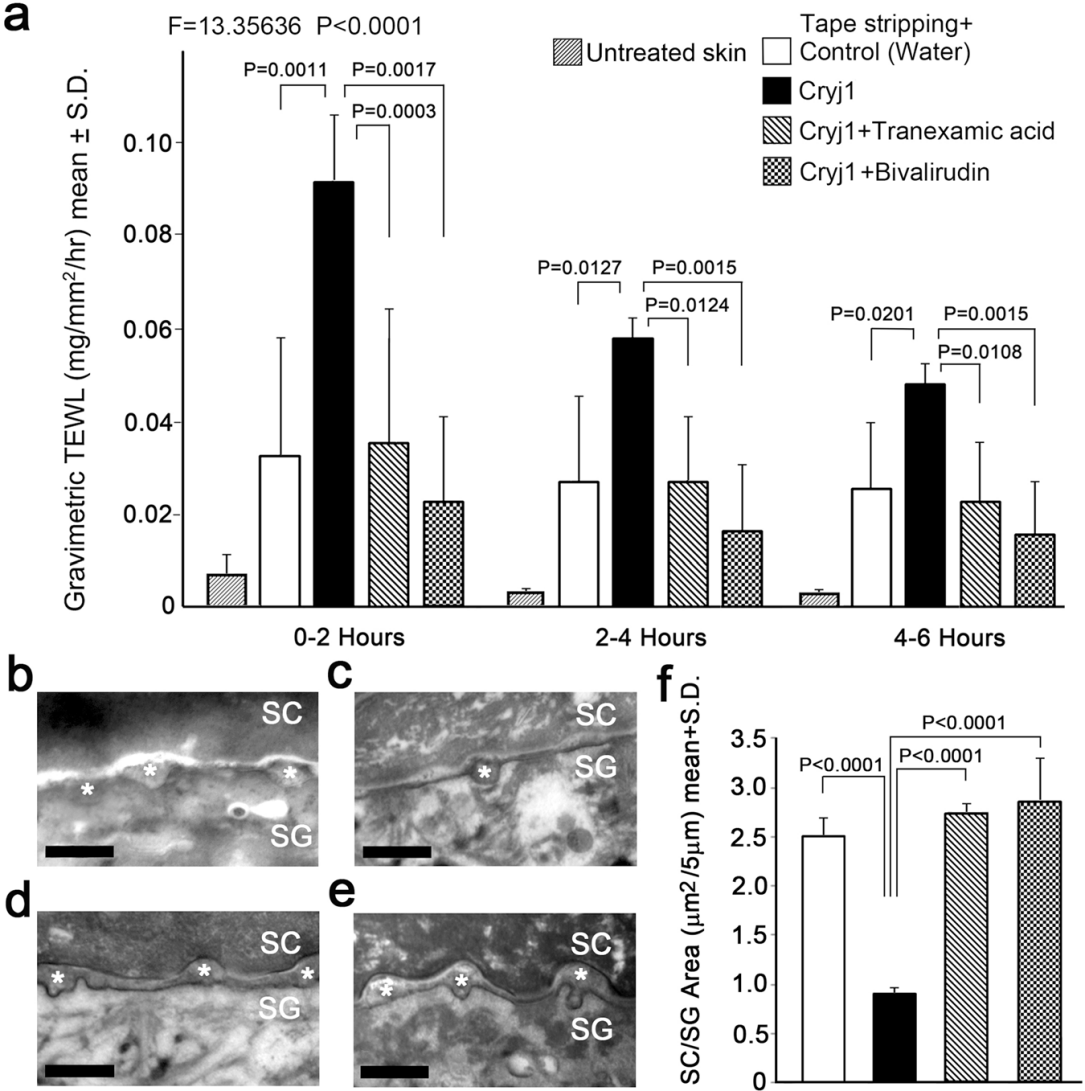
Evaluation of the water-impermeable barrier function of skin *ex vivo*, and electron-microscopic observation after application of Cry j1 with or without tranexamic acid or bivalirudin. (**a**) The vertical axis shows the amount of water loss during 0–2, 2–4 or 4–6 h after tape stripping (TS) (n = 5–6). Application of Cry j1 (1 μg/ml) dramatically increased the water loss. The Cry j1-induced increase was significantly reduced by concomitant application of tranexamic acid (100 mM) or bivalirudin (1 μg/ml). Bars and lines represent mean ± SD. (**b**–**f**) Electron-microscopic observation of *ex vivo* skin samples. (**b**) Tape-stripping (TS) 10 times and application of water. (**c**) TS and application of Cry j1 (1 μg/ml). (**d**) TS and application of Cry j1 (1 μg/ml) and tranexamic acid (100 mM). (**e**) TS and application of Cry j1 (1 μg/ml) and bivalirudin (1 μg/ml). SG: stratum granulosum, SC: stratum corneum. Asterisks indicated intercellular lipid domains. Bars = 500 nm. (**f**) Quantitative evaluation of stratum corneum/stratum granulosum (SC/SG) lipid domains. Intercellular lipid domains in tape-stripped skin were significantly reduced by the application of Cry j1. The effect of Cry j1 was blocked by co-application of tranexamic acid or bivalirudin.

The stratum corneum serves as a water-impermeable barrier, and when the barrier function is damaged by organic solvents, or by tape stripping, a series of homeostatic systems come into play^12^. First, exocytosis of lipid-containing granules, lamellar bodies, is accelerated and the lipids are secreted into the intercellular domain between the stratum granulosum and stratum corneum to reconstruct the water-impermeable membrane^12^. Calcium ion influx delays the barrier recovery process^13^. Thus, we evaluated the barrier recovery and observed the intercellular lipid domains by means of electron-microscopy. Topical application of Cry j1 after the barrier disruption of human skin in an *ex-vivo* system significantly delayed the barrier recovery, and concomitant application of either tranexamic acid or bivalirudin significantly blocked the delay (Fig. 4a). Electron-microscopic observations supported the results of the barrier study. After application of Cry j1 to tape-stripped skin, the intercellular domains between stratum granulosum (SG) and stratum corneum (SG) were significantly reduced. In contrast, when tranexamic acid or bivalirudin was applied together with Cry j1, thick intercellular lipid domains were observed between SG and SC (Fig. 4b–e). The results of quantitative evaluation are shown in Fig. 4f. These findings suggested that bivalirudin and tranexamic acid blocked the delay of the barrier recovery by inhibiting Cry j1-induced serine protease activity and calcium response.

## Discussion

Previous studies have shown that PAR-2 in keratinocytes plays an important role in epidermal permeability barrier homeostasis^14,15^. On the other hand, thrombin is a coagulation factor that plays a central role in the clotting cascade^4^, but it also has other biological functions, for example in muscle cells^16^, fibroblasts^17^ and monocytes^18^, some of which are mediated by PAR-1. In human keratinocytes, the thrombin/PAR-1 pathway promotes proliferation^8^, and it also participates in wound healing^8,19^. The results of the present study suggest that thrombin and PAR-1 or a PAR-1-like receptor might also play an important role in epidermal permeability homeostasis, at least in mediating the adverse effects of the cedar pollen antigen Cry j1. However, involvement of PAR-1 remains to be confirmed, as we cannot completely rule out the possibility of off-target effects of our PAR-1 siRNA mixture. Nevertheless, previous reports have demonstrated that the thrombin/PAR-1 pathway is involved in barrier homeostasis in endothelial cells^20,21^. Thus, it seems plausible that it might be involved in a variety of homeostatic processes.

In our protease activity assay, application of Cry j1 induced a temporary increase of protease activity. However, in our *ex vivo* study, topical application of Cry j1 delayed the barrier recovery process for a long period of time. A possible explanation of these apparently conflicting findings is that when Cry j1 is applied to human skin, it remains on the skin surface for a substantial period, resulting in a prolonged increase of protease activity, which in turn leads to impairment of barrier homeostasis.

In our previous study on Cry j1^1^, we found that a PAR-2-specific antagonist, FSLLRY-NH2_,_ decreased the Cry j1-induced increase of intracellular calcium level and blocked the delay of the barrier recovery induced by Cry j1 in an *ex-vivo* system. These results seemed to suggest that PAR-2 might play an important role in the adverse effects of Cry j1. However, the results of present study indicate that PAR-1 is also important. In this connection, it should be noted that FSLLRY-NH2 was reported to block the trypsin-induced intracellular calcium elevation of Kirsten virus-transformed rat kidney cells even though it did not inhibit trypsin activity, while the intracellular calcium elevation induced by PAR-2 agonist SLIGRL-NH2 was not blocked by FSLLRY-NH2^22^. It was concluded that the mechanism through which FSLLRY-NH2 blocked the activation of PAR-2 by trypsin was not inhibition of trypsin’s proteolytic activity, but rather an interaction with a tethered ligand receptor-docking site. However, the precise molecular mechanism of FSLLRY-NH2’s action is still unclear.

It was also reported that thrombin elicited ERK1/2 phosphorylation in mesenchymal stem cells and this action was potently inhibited by FSLLRY-NH2, although the PAR-1-specific antagonist SCH79797 was not inhibitory^23^. In our study, FSLLRY-NH2 significantly reduced the thrombin-induced calcium response in cultured keratinocytes (see Supplementary Fig. S4). Thus, it appears that FSLLRY-NH2 not only blocks PAR-2, but also inhibits the effects of thrombin. Further studies will be needed to establish the mechanisms involved.

Previous studies have shown that topical application of heparanoid, an inhibitor of thrombin, improves dry skin in patients with atopic dermatitis or senile xerosis^24,25^, possibly by acting as a moisturizer. However, our results raise the possibility that heparanoid might improve the skin condition by inhibiting thrombin.

Tranexamic acid improves epidermal hyperplasia and accelerates the barrier recovery induced by repeated barrier disruption^2^. In that study, an increase of protease activity in the epidermis after tape stripping was also observed^2^. The results of the present study suggest this might have been due to activation of thrombin. Tranexamic acid also inhibits melanogenesis^26^ and is effective against melasma^27^. Moreover, it improves rosacea^28^. Although the mechanisms of these phenomena have not been fully clarified, the results of the present study suggest a potential new strategy for treating a variety of skin diseases. In some cases, plasminogen from blood might play an important role and tranexamic acid might be effective as an inhibitor of plasminogen activation, while in other cases, such as skin diseases strongly associated with keratinocytes, tranexamic acid might effective as an inhibitor of some other type of protease.

In conclusion, tranexamic acid blocked the delay of the barrier recovery induced by Cry j1, apparently by inhibiting activation of thrombin. PAR-1 or a PAR-1-like receptor might also play a role in mediating the pathology induced by Cry j1.

## Materials and Methods

### Cells and cell culture

Normal human epithelial keratinocytes were purchased from Kurabo (Osaka, Japan) and cultured in EPILIFE-KG2 (Kurabo, Osaka, Japan). Keratinocytes were seeded onto collagen-coated glass coverslips (Matsunami, Osaka, Japan) and used within 4 days. Keratinocytes were first grown to 100% confluency in low-Ca^2+^ (0.06 mM) medium with or without siRNA for 24–48 h and then incubated in high-Ca^2+^ (1.8 mM) medium for 24–48 h.

### Materials

Cry j1 was purchased from Hayashibara Co., Ltd (Okayama, Japan), thrombin from Nakarai Tesque, Inc. (Tokyo, Japan), soybean trypsin inhibitor (SBTI) from Sigma-Aldrich (St. Louis, MO, USA), bivalirudin from ProSpec-Tany TechnoGene Ltd. (Rehovot, Israel), tranexamic acid from LKT Laboratories, Inc. (St. Paul, MN, USA), PAR-1 agonist TFLLR-NH2 from Tocris Bioscience (Bristol, UK), PAR-2 agonist SLIGKV-NH2 from Tocris Bioscience, Nonidet P-40 (MP40) from Nakarai Tesque, Inc. (Tokyo, Japan), and protein inhibitor cocktail from Sigma-Aldrich (St. Louis, MO, USA).

### Ratiometric fluorescence measurement of intracellular calcium

Changes of intracellular calcium concentration in single cells were measured with Fura-2 AM according to the manufacturer’s instructions (Molecular Probes Inc., OR, USA). Briefly, cells were loaded with 5 μM Fura-2 AM at 37 °C for 45 min. After loading, the cells were rinsed with balanced salt solution containing (in mM): NaCl 150, KCl 5, CaCl_2_ 1.8, MgCl_2_ 1.2, HEPES 25, and D-glucose 10 (pH 7.4), abbreviated as BSS(+), and incubated for a further 10 min at room temperature to allow de-esterification of the loaded dye.

The coverslip was mounted on an inverted epifluorescence microscope (ECLIPSE Ti, Nikon, Tokyo, Japan), equipped with a 75 W xenon lamp and band-pass filters of 340 and 380 nm. Imaging was done with a high-sensitivity CCD camera (ORCA-R2, Hamamatsu Photonics, Hamamatsu, Japan) under the control of a Ca^2+^ analyzing system (AQUACOSMOS/RATIO, Hamamatsu Photonics). The intracellular calcium concentration was measured every second.

### Protease assay

For the assay of protease activity, an AmpliteTM Universal Fluorimetric Protease Activity Assay Kit, Green Fluorescence (AAT Bioquest, Sunnyvale, CA, USA) was used. To examine the time course of protease activity in the medium with or without SBTI (final concentration 1 μM), bivalirudin (final concentration 100 ng/ml) or tranexamic acid (final concentration 10 mM), we added 180 μl of the AmpliteTM kit substrate (fluorescent casein conjugate, diluted 1:100) to a culture of differentiated human keratinocytes in 14 mm dishes, then added 20 μl of BSS(+) solution with/without Cry j1 (100 ng/ml). The fluorescence (excitation filter of 455–485 nm and emission filter of 500–545 nm) was imaged with a high-sensitivity CCD camera (ORCA-R2, Hamamatsu Photonics, Hamamatsu, Japan) under the control of a Ca^2+^ analyzing system (AQUACOSMOS/RATIO, Hamamatsu Photonics).

### Human skin tissue culture

Human tissues were purchased from Biopredic International (Rennes, France) via KAC Co., Ltd. (Kyoto, Japan). The samples had been obtained following plastic surgery, with informed consent. The excised skin was dermatomed to 340–440 μm thickness (containing epidermis and dermis), and then discs (10 mm in diameter, thickness about 2 mm) were punched out and transferred to our laboratory. Four samples of skin tissues from abdomen of healthy, independent subjects (30–50 years old, Caucasian females) were used for the study. This study was approved by the ethics committee of Shiseido in accordance with the guidelines of the National Institute of Health. Tissues were cultured in long-term skin culture medium (LTSC medium), provided by Biopredic International.

### Transepidermal water loss

Gravimetric transepidermal water loss (TEWL) was measured as described previously^29^. Skin sections were placed dermis-side-down onto glass-based dishes and the lateral edges and dermal surface were sealed with petrolatum, so that water loss could occur only through the epidermal surface. The stratum corneum was stripped 20 times with adhesive tape, and then Cry j1 with/without other reagents was immediately applied for 20 min. Skin sections were kept at ambient temperature (37 °C) and humidity (30–35%), and weighed every 2 h. TEWL levels are reported as milligrams of water lost per square millimeter per hour. Skin sections from four different subjects were used.

### Electron-microscopic observation

Skin samples for electron microscopy were minced (<0.5 mm^3^ pieces), fixed overnight in modified Karnovsky’s fixative, and then post-fixed in 2% aqueous osmium tetroxide or 0.2% ruthenium tetroxide as described previously^30^. After fixation, all samples were dehydrated in graded ethanol solutions, and embedded in an Epon-epoxy mixture. Stratum corneum/stratum granulosum (SC/SG) lipid domains were quantified using osmium post-fixed material. We used 4 samples from different subjects for each condition. Measurements were made without knowledge of the prior experimental treatment. The area measurements were made from photographs of randomly selected sections at a constant magnification, using computer software (NIH Image).

### Quantitative real-time PCR (RT-PCR)

Total RNA from human keratinocytes was isolated using an RNeasy mini kit (QIAGEN, Hilden, Germany). Complementary DNA (cDNA) synthesis from 1 μg of total RNA was performed using SuperScript VILO Master Mix (Invitrogen, Carlsbad, USA). The PCR reactions were performed using LightCycler 480 Probes Master (Roche, Basal, Switzerland), cDNA and specific primer pairs: GUSB: forward, cgccctgcctatctgtattc and reverse, tccccacagggagtgtgtag; Plasminogen and plasmin: forward, gaacaattggctcccacag and reverse, ggatgtgcctcggtagctc; PAR-1 (*F2R*): forward, aaaatggatacctgctctag and reverse, gaatcctcaggttattcact; PAR-2 (*F2RL1*): forward, tgctagcagcctctctctcc and reverse aaggcttcttcctttagaggatct; PAR-3 (*F2RL2*): forward, aaatcactccactgcttac and reverse, cctttgaagcatatttctta; PAR-4 (*F2RL3*) forward, aacctctatggtgcctacgt and reverse, ggccgacacgtagtagta, on an LightCycler 480 Sytem II (Roche, Basel, Switzerland). Results were normalized with the GUSB gene.

### siRNA transfection

Human keratinocytes were grown to 80% confluency, and transfected with 10 nmol of the following siRNA mixtures, using the transfection reagent RNA iMAX (Thermo Fisher Scientific, Waltham, USA) in serum-free and antibiotic-free EpiLife, OptiMen (Thermo Fisher Scientific), as described in the manual. Scramble control: ugguuuacaugucgacuaa, ugguuuacauguuguguga, ugguuuacauguuuucuga and ugguuuacauguuuuccua. PAR-1 *(F2R*): gaaaguggguuaacugaau, ggcaguugauggcaaguaa, cggcagugauuggcaguuu and cauaagcauugaccgguuu. PAR-2 *(F2RL1*): gguauugggucaucgugaa, guuaagaccuccuauugag, cuaguaaccuucugcuugu and gugguggguuugccaagua. PAR-3 *(F2RL2*): gcuuagauccauuccuuua, agaugguugugguauguua, ggcauucuuuggauucuua and gccaucauccggacacuua. PAR-4 *(F2RL3*); ccucuuugcuccagugaca acuacuacgugucggccga, ggggaaggcuguacugggu and ucaccugccuggcgcuguu. prothrombin (*F2*): cgggaagccuggcgacuuu, cugcaugucuggaagguaa, gaaggucauugaucaguuu and guaccagacuuucuucaau (GE Dharmacon, Lafayette, USA) or tacaagcctgatgaagggaaa, and cagcgaggacgcctcgagata (QIAGEN, Hilden, Germany).

### Enzyme-linked immunosorbent assay (ELISA)

Human keratinocytes were harvested in MP40 buffer containing Tris-HCl (pH 7.5) 50 mM, NaCl 150 mM, MP40 2% and protein inhibitor cocktail 1% and homogenized in a glass homogenizer. The lysate was centrifuged (15,000 rpm, 30 min), and thrombin in the supernatant was measured with a Human Thrombin ELISA kit (abcam, Cambridge, UK) according to the manufacturer’s protocol. Samples were analyzed four times, and the average value was taken.

### Statistics

Statistical significance of differences among three or more groups was determined by ANOVA with Scheffé's method. P < 0.05 was considered significant. Student’s t test was used to determine the significance of differences between two groups.

## Electronic supplementary material

Supplementary information
