# Supplementary material for: Tranexamic acid blocks the thrombin-mediated delay of epidermal permeability barrier recovery induced by the cedar pollen allergen, Cry j1
Source: Sci Rep. 2018 Oct 23;8:15610. doi: 10.1038/s41598-018-33898-7 (PMC6199325; doi:10.1038/s41598-018-33898-7)
Supplement: Supplementary file 1 — Supplementary information [file 41598_2018_33898_MOESM1_ESM.pdf]

Tranexamic acid blocks the thrombin-mediated delay of epidermal permeability barrier recovery induced by the cedar pollen allergen, Cry j1

*Nakanishi S[1], Kumamoto J[2], Denda M[1]

[1] Shiseido Research Center, Yokohama, Japan

[2] Research Institute for Electronic Science, Hokkaido University, Sapporo, Japan

*Corresponding author: Shinobu Nakanishi, Shiseido Global Innovation Center, 2-2-1, Hayabuchi, Tsuzuki-ku, Yokohama, 224-8558, Japan. E-mail address: shinobu.nakanishi@to.shiseido.co.jp

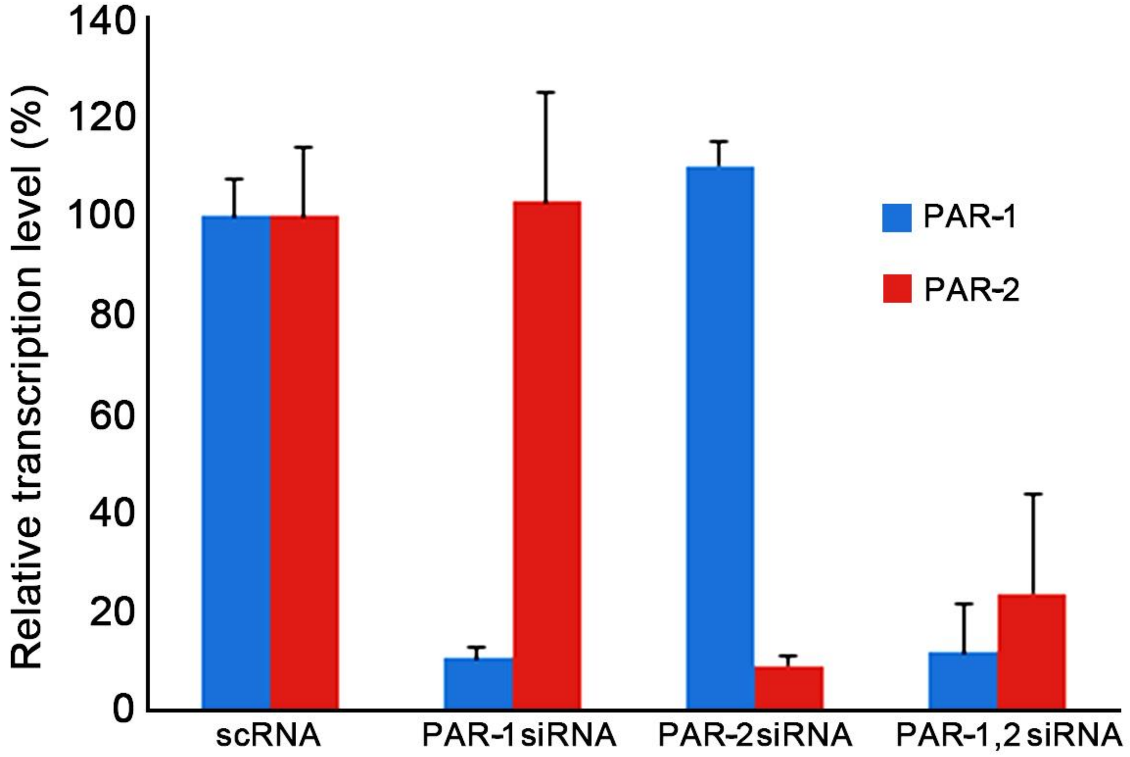

Supplementary Fig. S1. Transcription levels of PAR-1 and PAR-2 in keratinocytes treated with PAR-1 siRNA, PAR-2 siRNA, or both. RT-PCR showed significantly lower PAR-1 mRNA levels in the PAR-1 siRNA-treated cells and the PAR-1 plus PAR-2 siRNA-treated cells than in the scramble RNA-treated cells or the PAR-2 siRNA-treated cells (blue bar). Similarly, significantly lower PAR-2 mRNA levels were observed in the PAR-2 siRNA-treated cells and the PAR-1 plus PAR-2 siRNA-treated cells than in the scramble RNA-treated cells or the PAR-1 siRNA-treated cells (red bar). The results were obtained in three independent experiments. Bars and lines represent mean ± SD.

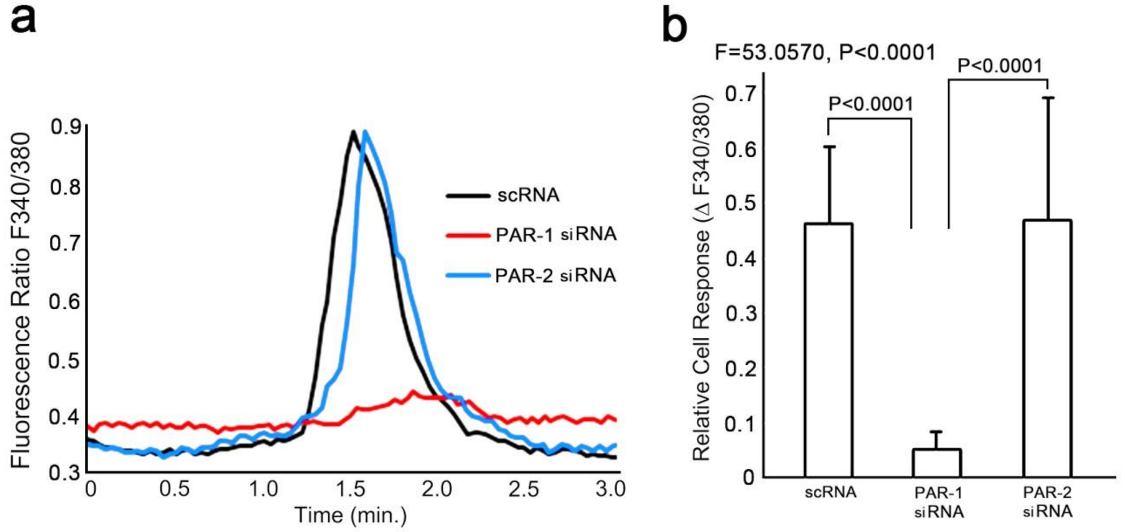

Supplementary Fig. S2. Calcium response induced by thrombin in PAR-1 or PAR-2 siRNA-treated keratinocytes. (**a**), Application of thrombin (100 pg/ml) to the scramble RNA-treated cells (black line) and PAR-2 siRNA-treated cells (blue line) increased the level of intracellular calcium by approximately 0.46 unit and 0.47 unit respectively, while application of the same amount of thrombin to the PAR-1 siRNA-treated cells (red line) increased the level of intracellular calcium by approximately 0.05 unit. (**b**), Quantitation of fluorescence ratio change after application of thrombin to the cells treated with scramble RNA, PAR-1 siRNA and PAR-2 siRNA (n = 20 cells). Similar results were obtained in three independent experiments. Bars and lines represent mean ± SD.

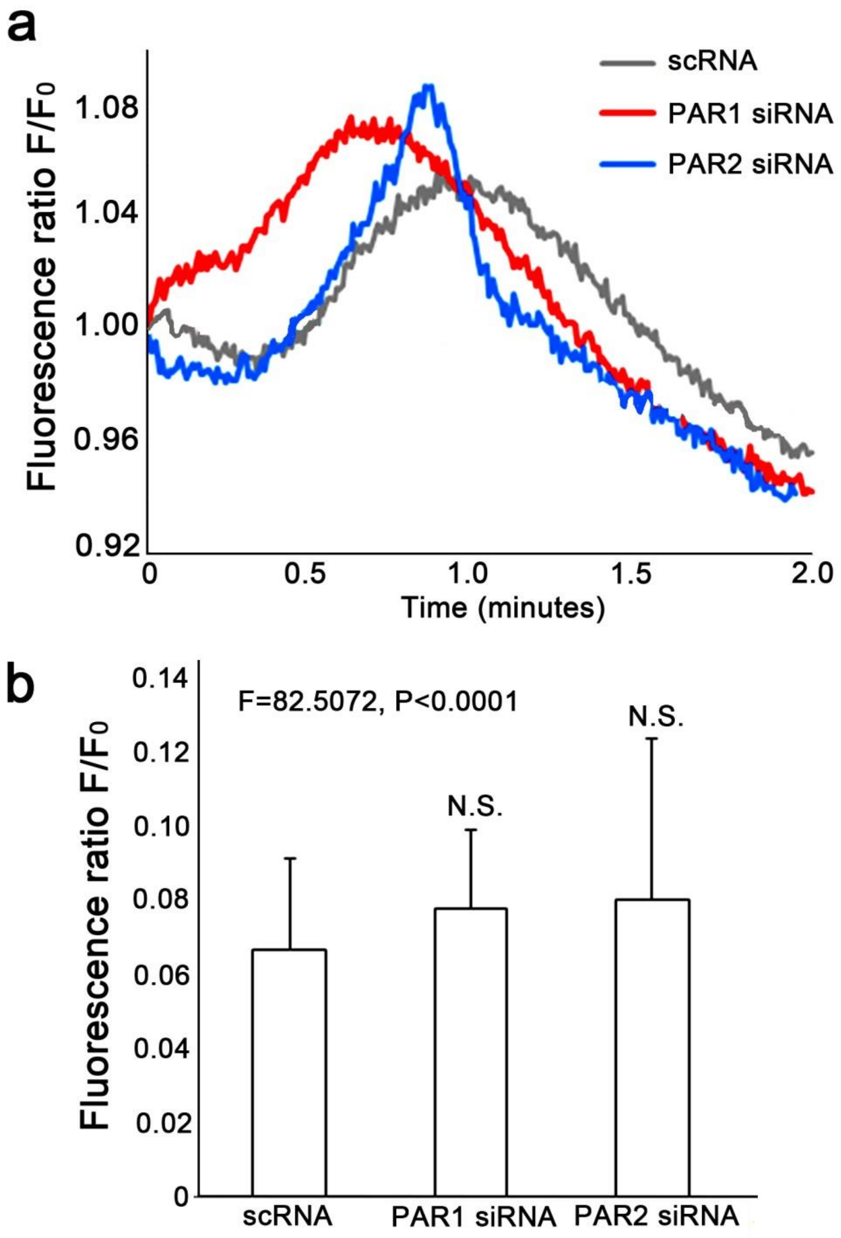

Supplementary Fig. S3. Protease activity induced by Cry j1 in PAR-1, PAR-2 or thrombin siRNA-treated keratinocytes. (**a**), Application of Cry j1 (100 ng/ml) to the cells treated with scramble RNA, PAR-1 siRNA or PAR-2 siRNA at time 0 s induced a rapid, transient increase of protease activity (gray, red and blue line). The vertical scale is normalized by the fluorescence at time 0. (**b**), Quantitation of fluorescence change within 1 min after application (n = 30-50 cells). No significant difference was observed among the scramble siRNA-treated, PAR-1 siRNA-treated and PAR-2 siRNA-treated cells. Similar results were obtained in three independent experiments. Bars and lines represent mean ± SD.

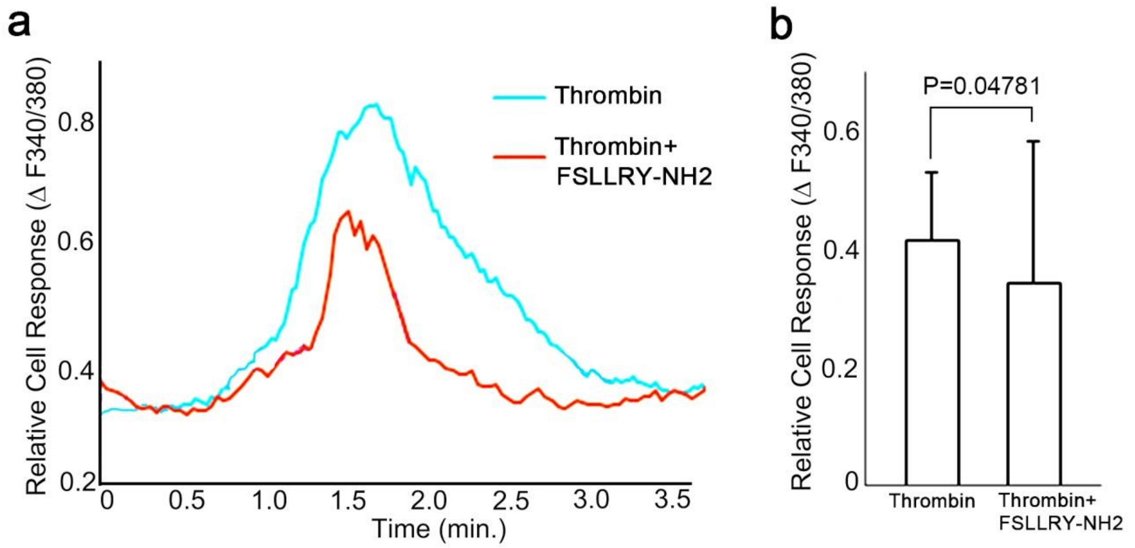

Supplementary Fig. S4. Calcium response of keratinocytes treated with thrombin. (**a**), Application of thrombin (100 pg/ml) increased the level of intracellular calcium by approximately 0.41 unit (cyan line), while application of the same amount of thrombin with FSLLRY-NH2 (100 μM) (red line) increased the level of intracellular calcium by approximately 0.34 unit. (**b**), Quantitation of fluorescence ratio change after application of thrombin with or without FSLLRY-NH2 (n = 20 cells). Similar results were obtained in three independent experiments. Bars and lines represent mean ± SD.
